# The Protection of Polysaccharide from the Brown Seaweed *Sargassum graminifolium* against Ethylene Glycol-Induced Mitochondrial Damage

**DOI:** 10.3390/md11030870

**Published:** 2013-03-13

**Authors:** Chao-Yan Zhang, Wen-Hui Wu, Min-Bo Lan

**Affiliations:** 1 Shanghai Key Laboratory of Functional Materials Chemistry, Research Centre of Analysis and Test, East China University of Science and Technology, Shanghai 200237, China; E-Mail: chyzhang@shou.edu.cn; 2 College of Food Science and Technology, Institutes of Marine Sciences, Shanghai Ocean University, Shanghai 201306, China; E-Mails: kongting072@163.com (T.-K.); whwu@shou.edu.cn (W.-H.W.)

**Keywords:** polysaccharide, mitochondrial damage, hyperoxaluric, urinary stones, reactive oxygen species (ROS)

## Abstract

The aim of the present study is to evaluate the protective effect of polysaccharide from the Brown Seaweed *Sargassum graminifolium* (SGP) on ethylene glycol-induced kidney damage and the mechanism of SGP-mediated protection. Mitochondrial lipid peroxidation, mitochondrial swelling, the activity of succinate dehydrogenase (SDH), ATPases and mitochondrial antioxidant enzymes was observed in hyperoxaluric rats. Administration of SGP (25, 100 and 400 mg·kg^−1^, intragastrically) increased the activities of antioxidant enzymes, SDH and Na^+^/K^+^-ATPases, Ca^2+^-ATPases, Mg^2+^-ATPases, also decreased mitochondrial lipid peroxidation and mitochondrial swelling. SGP exhibited a protective effect by improving antioxidant enzymes and restoring mitochondrial dysfunction in the kidney of hyperoxaluric rats. It may be used as a promising therapeutic agent to provide superior renal protection.

## Abbreviations

SGP: polysaccharide from *Sargassum graminifolium*

Ox: oxalate

CaOx: calcium oxalate

SDH: succinate dehydrogenase

MDA: malondialdehyde

ROS: reactive oxygen species

EG: ethylene glycol

AC: ammonium chloride

ATP: adenosine triphosphate

SOD: superoxide dismutase

GSH-PX: glutathione peroxidase

CAT: catalase

## 1. Introduction

In recent years, there has been much interest in isolating novel bioactive compounds with beneficial effects on human health from marine resources. Marine algae are valuable sources of structurally diverse bioactive compounds. Sulfated polysaccharides are widespread in marine algae, especially brown seaweeds. Sulfated polysaccharides show various biological activities, including anticoagulant, antioxidant, antiviral, anticancer and immunoregulation activities [1,2,3,4,5,6].

It is widely known that urinary stones pathogenesis is multifactorial; the mechanism of renal calcium crystallization remains unclear [7,8,9]. Recent research has shown that exposure of renal cells to high concentrations of Ox and/or calcium oxalate (CaOx) crystals leads to the production of reactive oxygen species (ROS) in tissue culture and animal model studies [10,11,12]. Studies have shown that mitochondria are major sources of intracellular ROS, because they are sites of aerobic metabolism, spaces for the storage and supply of energy [13]. The primary function of the mitochondrial electron transport chain is the production of cellular ATP by oxidative phosphorylation. On the other hand, mitochondria are highly sensitive to free radical damage or attack, because of the relatively high content of polyunsaturated fatty acids in their membranes [14]. Hence, mitochondria might serve as a source as target for reactive species [15].

Some studies also show that mitochondrial dysfunction is the key event in the pathogenesis of kidney stones and its amelioration might prevent renal damage in stone formation. Research had suggested administration of fucoidan to normalize the redox status of the renal cells under hyperoxaluria, and also protect the mitochondrial damage [16].

Brown seaweeds have been used in traditional Chinese medicine for more than 1000 years [17]. *Sargassum graminifolium*, a brown seaweed extensively distributed along the coasts of the South China Sea and the East China Sea, is commonly consumed as seafood and has antiallergic effects and some other medical properties [18]. Therefore, it is important to evaluate the polysaccharide bioactivity of *S. graminifolium* to fully utilize this rich resource. Our previous reports have shown the sulfated polysaccharide (SGP) from the brown seaweed *Sargassum graminifolium* was able to inhibit calcium oxalate crystallization and its antioxidant properties *in vitro* [19].

Hence, the present study is targeted to evaluate the protective effect of SGP on ethylene glycol-induced kidney damage by analyzing the relationship between SGP and the mitochondria. Also, the present study is an attempt to explore whether supplementation of SGP, a sulfated polysaccharide, could ameliorate the mitochondria dysfunction.

## 2. Results and Discussion

### 2.1. Characterization of SGP

**Figure 1:**
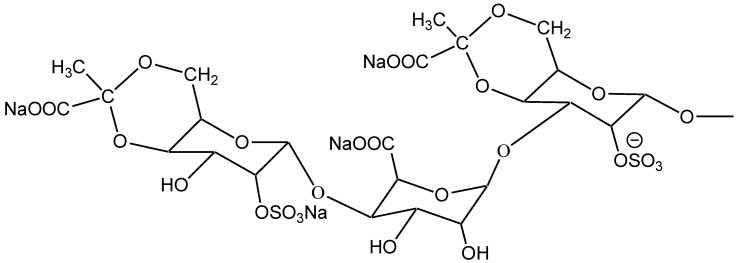
Chemical structure of the repeating units of *Sargassum graminifolium* (SGP).

Judging from the electrophoretogram, it migrated as a single band on the cellulose acetate membrane showing that SGP is a kind of homogeneous polysaccharide. Furthermore, the molecular weight of SGP is 11,887 Da on the basis of its ESI-MS; and the composition of SGP was identified with thin-layer chromatography and mass spectra. The chemical structure of the repeating units of SGP is displayed in Figure 1.

### 2.2. Effects of SGP on Mitochondrial Lipid Peroxidation

**Figure 2:**
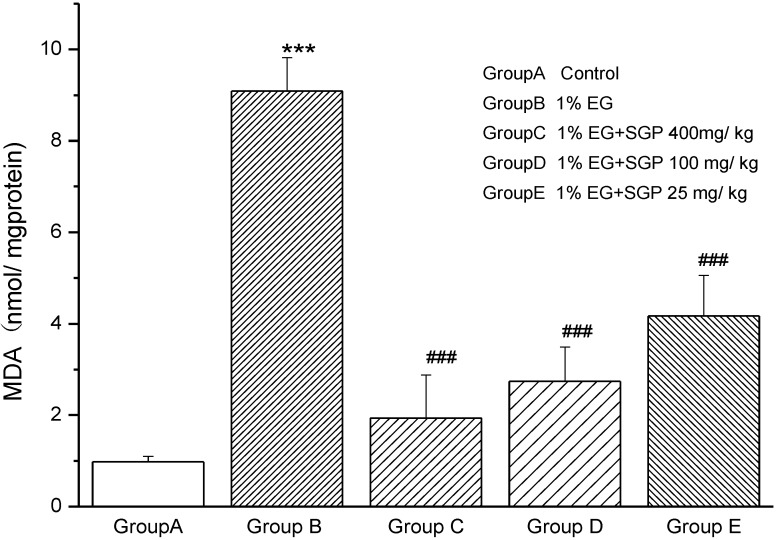
Effect of SGP on mitochondrial lipid peroxidation in experimental hyperoxaluria rats. Values are expressed as mean ± S.D. for 6 animals in each group. Comparisons are made between: ^#^—Group B *vs.* Groups C, D or E; *—Group A *vs.* Groups B. ^#^ *P* < 0.05, ^##^ *P* < 0.01, ^###^ *P* < 0.001; * *P* < 0.05, ** *P* < 0.01, *** *P* < 0.001.

Figure 2 shows the MDA levels in mitochondrial fraction of different experimental groups (*P* < 0.001). Group B rats showed 9.3-fold increase in MDA compared with Control (Group A), which indicated that lipid peroxidation was very strong because of Ethylene glycol and ammonium chloride induced urolithiasis. MDA levels decreased with the SGP dose increased. The level of MDA with administration of high dose SGP was similar to Control Group A.

### 2.3. Effects of SGP on Mitochondrial Swelling

**Figure 3:**
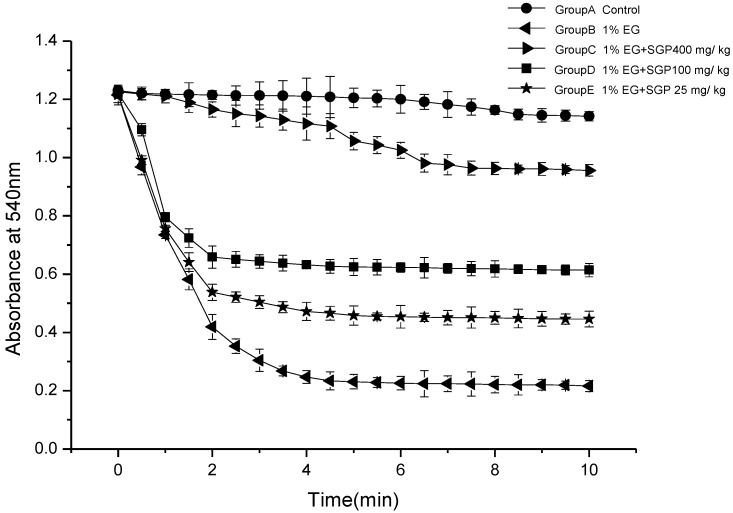
Mitochondrial swelling in hyperoxaluria and the effect of SGP.

Figure 3 demonstrates mitochondria swelling degree of different experimental groups. It was indicated that mitochondrial fraction from urolithiasis rats significantly swell compared with control under the experimental conditions employed. It was demonstrated that Ethylene glycol and ammonium chloride induced urolithiasis damage to the kidney mitochondrial membrane function of the rats. Administration of SGP to rats was able to reduce the mitochondrial swelling. High dose SGP and middle dose SGP decreased significantly (*P <* 0.05) compared with ethylene glycol group, especially high dose SGP group of absorbance change curve similar to Control Group. This indicates that SGP in the 100 mg/kg–400 mg/kg range can effectively reduce the hyperoxaluric rats swelling degree and that there are certain dose-effect relationships.

### 2.4. Effects of SGP on SDH Content of Mitochondrial

**Figure 4:**
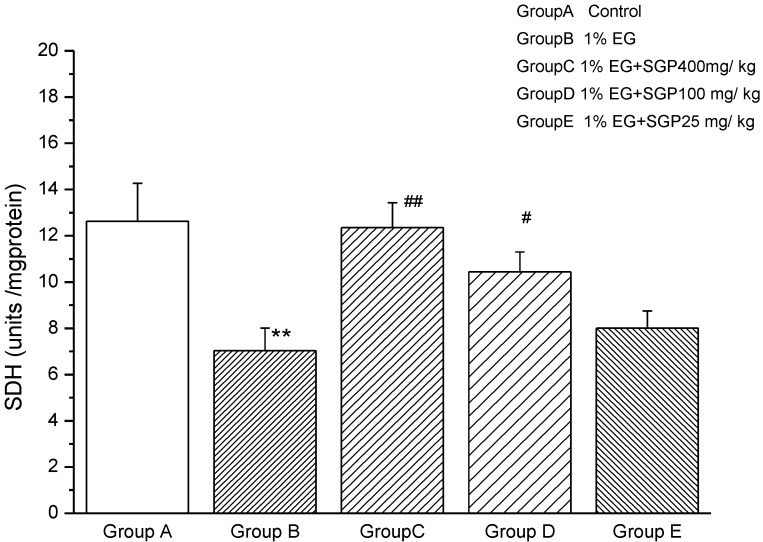
Effect of SGP on mitochondrial SDH activity in experimental hyperoxaluria rats. Values are expressed as mean ± S.D. for 6 animals in each group. Comparisons are made between: ^#^—Group B *vs.* Groups C, D or E; *—Group A *vs.* Groups B. ^#^ *P* < 0.05, ^##^ *P* < 0.01;* *P* < 0.05, ** *P* < 0.01, *** *P* < 0.001.

Figure 4 shows that SDH activity decreased significantly (*P* < 0.01) from Ethylene glycol induced hyperoxaluric rats compared with control, demonstrating that the kidney mitochondrial of rats were damaged, affecting aerobic metabolism—three tricarboxylic acid cycle function. Administration of SGP to hyperoxaluric rats was able to increase SDH activity, high dose SGP and middle dose SGP increased significantly (*P* < 0.01 or *P* < 0.05) compared with ethylene glycol group. This was explained SGP in the 100 mg/kg–400 mg/kg range was effective to improve SDH activity and increasing concentrations of SGP resulted in increased SDH activity of rats.

### 2.5. Effects of SGP on Mitochondrial ATPases

Table 1 shows ATPases levels in various experimental groups. It was found that Na^+^/K^+^-ATPases, Ca^2+^-ATPases, Mg^2+^-ATPases activity decreased significantly (*P* < 0.001 or *P* < 0.01) from Ethylene glycol induced hyperoxaluric rats compared with control, demonstrating that the kidney mitochondrial of rats were damaged, affecting Energy metabolism of rats kidney mitochondrial. Administration of SGP to hyperoxaluric rats was able to increase ATPases activity, high dose SGP and middle dose SGP increased Na^+^/K^+^-ATPases, Ca^2+^-ATPases, Mg^2+^-ATPases activity significantly (*P* < 0.01 or *P* < 0.05) compared with ethylene glycol group; Furthermore, Low dose SGP too increased Ca^2+^-ATPases significantly (*P* < 0.05).

**Table 1:** Effect of SGP on mitochondrial ATPase activity in experimental Hyperoxaluria.

Parameters	Group A	Group B	Group C	Group D	Group E
Na^+^/K^+^-ATPases	3.05 ± 0.23	1.36 ± 0.07 ***	2.97 ± 0.15 ^##^	2.32 ± 0.29 ^#^	1.47 ± 0.18
Ca^2+^-ATPases	2.25 ± 0.19	1.05 ± 0.03 **	2.18 ± 0.16 ^##^	2.01 ± 0.21 ^##^	1.96 ± 0.13 ^#^
Mg^2+^-ATPases	2.88 ± 0.17	0.90 ± 0.04 ***	2.69 ± 0.0 ^###^	2.12 ± 0.11 ^##^	1.24 ± 0.12

Units: μmol Pi h^−1^ mg protein^−1^. Values are expressed as mean ± S.D. for 6 animals in each group. Comparisons are made between: ^#^—Group B *vs.* Groups C, D or E; *—Group A *vs.* Groups B. ^#^ *P* < 0.05, ^##^ *P* < 0.01, ^###^ *P* < 0.001; * *P* < 0.05, ** *P* < 0.01, *** *P* < 0.001.

### 2.6. Effects of SGP on Mitochondrial Antioxidant Enzymes

Table 2 shows the altered activities of SOD, GSH-PX and CAT in various experimental groups. SOD, GSH-PX and CAT activities in ethylene glycol group decreased by 37.41%, 55.39% and 52.03%, compared with the control group, respectively. Further, GSH-PX and CAT activity in group C, D, E were significantly higher than those in group B (*P* < 0.05 or *P* < 0.01).The higher the dose, GSH-PX, CAT enzyme activity the closer the control group. And except SOD level in group E were near to group B, SOD level in group C and group D were significantly higher than those in group B (*P* < 0.05). This was suggested that administration of SGP significantly increased the activities of antioxidant enzymes.

**Table 2:** Effect of SGP on mitochondrial antioxidant enzymes levels in experimental Hyperoxaluria.

Parameters	Group A	Group B	Group C	Group D	Group E
SOD (U/mg prot)	58.22 ± 8.33	36.44 ± 4.94 **	53.46 ± 3.58 ^#^	43.33 ± 2.0 ^#^	36.62 ± 2.66
GSH-Px (U/mg prot)	35.24 ± 2.13	15.72 ± 1.47 **	32.76 ± 3.05 ^##^	25.29 ± 2.58 ^#^	18.55 ± 1.72 ^#^
Catalate (U/mg prot)	31.58 ± 0.94	15.15 ± 1.17 **	18.76 ± 0.93 ^#^	21.43 ± 0.67 ^#^	27.07 ± 0.73 ^#^

Values are expressed as mean ± S.D. for 6 animals in each group. Comparisons are made between: ^#^—Group B *vs.* Groups C, D or E; *—Group A *vs.* Groups B. ^#^ *P* *<* 0.05, ^##^ *P <* 0.01;* *P <* 0.05, ** *P* *<* 0.01.

### 2.7. Discussion

It has been found that the hyperoxaluria is closely related to the formation of kidney stones and mitochondrial dysfunction was one of the key factors [16]. ROS generated by the hyperoxaluria can cause tubular epithelial cells mitochondrial functions damage, and mitochondrial energy metabolism and SDH enzyme vitality being changed are the most important reasons for this damage [15,16,20]. In our research, the result show that SGP is effective for improving the SDH activity and ATPases levels, which is the first meaningful discovery for the mechanisms of SGP protection.

In normal conditions, generation of reactive oxygen species by mitochondria is a normal process as a consequence of existence. However, under pathological conditions, the development of tissue injury probably depends on the balance of the generation of reactive oxygen species and the tissues antioxidant defense mechanism. Complexes of free radical scavenging enzymes, including SOD, CAT and GSH-PX, have evolved to prevent excessive oxidant stress [21,22]. Studies of Alagarraju Muthukumar indicated that mitochondrial dysfunction resulting from GSH depletion could be a contributing factor in the development of calcium oxalate stones [23]. In our previous experiments [19], SGP showed favorable antioxidant activity in terms of its ability to scavenge superoxide radicals and DPPH, and showed significant reducing power *in vitro*. Further experiments *in vivo* showed that administration of SGP to hyperoxaluric rats is effective in decreasing the oxidative stress, by increasing the activities of antioxidant enzymes like SOD, GSH-PX, CAT and limiting lipid peroxidation. This may be another mechanism for SGP protection.

The integrity of mitochondrial membrane is important for maintaining the mitochondrial function. It has been found that mitochondrial permeability transition pore opening can induce the initial process of renal calcium crystallization [13]. Some studies have shown that exposure in the environment of calcium oxalate crystals can increase mitochondrial swelling; the reason being that oxalate directly interacts with the inner mitochondrial membrane and then alters its permeability [24]. Hence, the integrity of membranes could be measured through swelling studies. Our studies also showed SGP can effectively reduce the mitochondrial swelling degrees in hyperoxaluric rats, which may well be the third mechanism of SGP protection.

It was reported that the sulfated polysaccharides extracted from edible seaweed *Fucus vesiculosus* have renoprotective effects in experimental hyperoxaluria, which can decrease reactive oxygen species, lipid peroxidation and mitochondrial swelling, and can increase the activities of antioxidant enzymes levels [25]. This is similar to our findings.

## 3. Experimental Section

### 3.1. Chemicals and Reagents

The kits for assay of Malondialdehyde (MDA) content, Succinate dehydrogenase (SDH), ATPases, superoxide dismutase (SOD), glutathione peroxidase (GSH-PX) and catalase (CAT) activity were purchased from NanJing JianCheng (NanJing JianCheng Bio Inst, China). Tissue Mitochondria Isolation Kits were purchased from Boyotime (Haimen, Boyotime Institute of Biotechnology). All the chemicals used were of analytical grade available. 

### 3.2. Plant Material

Briefly, the degreased *Sargassum graminifolium* powder(1kg) was incubated in a water bath at 90 °C for 3 h, and the residue was re-extracted twice and then concentrated to one-third of the original volume at 80 °C, adding 95% ethanol to the water extract until the ethanol concentration reached 80%. After standing overnight, the mixture was centrifuged at 2775 g for 15 min. The precipitate containing crude polysaccharides was washed with 95% ethanol, then with ethyl ether, and finally with acetone. Proteins were removed by adding trichloroacetic acid and centrifuging the mixture at 2775 g for 15 min. The resulting products were concentrated and then freeze-dried to get a dried slight brown white crude polysaccharide (8.06% yield). The crude powder was redissolved in distilled water and decolorized by H_2_O_2_. The better condition was: pH = 6, H_2_O_2_ 7%, temperature 40 °C, decoloring-time 3 h. At last, the second alcohol precipitation and washing process were applied to the decolorized solution. A white polysaccharide puriﬁed from *Sargassum graminifolium* was gotten (1.02% yield) and named SGP being used in this study.

SGP was identified by mass spectrometry, electrophoresis, ultraviolet spectroscopy, infrared spectroscopy, and thin-layer chromatography. Electrospray ionization (ESI) mass spectra were obtained on an Agilent G1969A LCMS-TOF. 

### 3.3. Animals

The male Wistar rats (200 ± 20 g) were acclimated for 7 days in cages before experiments under a controlled temperature of 22 ± 2 °C and were kept under a controlled 12 h light/dark cycle. Animals were given standard diet.

### 3.4. Ethylene Glycol-Induced Mitochondrial Damage in Rats

The thirty rats were randomly divided into five groups consisting of six rats each group. Group A rats served as vehicle-treated control. Group B rats received ethylene glycol in drinking water (containing 1% [v/v] ethylene glycol (EG) and 1% [w/v] ammonium chloride (AC)) in order to promote hyperoxaluria and CaOx deposition in the kidneys for 7 days to induce hyperoxaluria. Group C,D,E served as the drug control and received SGP, dissolved in saline (400 mg/kg, 100 mg/kg and 25 mg/kg body weight, respectively, intragastrically) every day from the 8th day up to the end of the experimental period [16,26].

### 3.5. Preparation of Mitochondria

At the end of 21 days, the rats were sacrificed and the kidneys were removed from the rats. Mitochondria were isolated as the introduction of Tissue Mitochondria Isolation Kit described.

The kidneys were homogenized 3–4 min in an ice-cold buffer with a homogenizer and centrifuged at 600× *g* for 5 min. The supernatant was further centrifuged at 11,000× *g* for 10 min in a new tube and then the supernatant removed from the tube, the precipitate as mitochondria pellet. 900 μL ice physiological saline and 200 μL lysine was added to the mitochondria pellet for the biochemical studies. All operations were performed at 4 °C.

### 3.6. Mitochondrial Lipid Peroxidation

The rats kidney level of MDA were analyzed using MDA kit from NanJing JianCheng (NanJing JianCheng Bio Inst, China) and the protocols was all followed the introduction in the kit. MDA served as the index of lipid peroxidation. MDA formed products with thiobarbituric acid. The red product formed gave an absorption maximum at 532 nm.

### 3.7. Measurement of Mitochondrial Swelling

The swelling extent of mitochondria was evaluated according to the decreased values of 520 nm absorption [16]. The suspension in mitochondrial swelling medium contained 10 mM HEPES (pH 7.4), 71 mM sucrose, 215 mM mannitol and 10 mM succinate. First, the mitochondrial pellet was kept in the suspension on ice until swelling; second, the suspension was monitored at 520 nm continuously for 10 min.

### 3.8. Assay of the Activity of the Succinate Dehydrogenase (SDH)

The rats kidney activity of SDH was analyzed using SDH kit from NanJing JianCheng (NanJing JianCheng Bio Inst, China), and the protocols were all followed the introduction in the kit.

### 3.9. Assay of activities of ATPases

The rats kidney level of ATPases were evaluated using ATPases kit from NanJing JianCheng (NanJing JianCheng Bio Inst, China), and the protocols were all followed the introduction in the kit. The method is based on that ATP can be decomposed into ADP and inorganic phosphorus. Determination of inorganic phosphorus content can calculate ATPases activity.

### 3.10. Mitochondrial Antioxidant Enzymes

The rats kidney activities of enzymes (GSH-PX, CAT and SOD) were analyzed using kits from NanJing JianCheng (NanJing JianCheng Bio Inst, China), and the protocols were all followed according to the instructions in the kit. All antioxidant enzyme activities were determined by spectrophotometer.

### 3.11. Statistical Analysis

The results are expressed as mean ± standard deviation (S.D.) for six animals in each group. Differences between groups were assessed by one way analysis of variance (ANOVA) with *post hoc* Dunnett’s test or by Student’s *t*-test using the SPSS software package for Windows. Significance at *P*-values < 0.001, <0.01, <0.05 have been given respective symbols in the figures and tables.

## 4. Conclusions

In conclusion, our results indicate that SGP prevents ethylene glycol-induced mitochondrial damage in hyperoxaluria rats. In addition, the mechanism of SGP-mediated protection may be due to the increased activities of antioxidant enzymes, SDH and Na^+^/K^+^-ATPases, Ca^2+^-ATPases, Mg^2+^-ATPases, and the decreased mitochondrial swelling and lipid peroxidation. Therefore SGP may be used as a promising therapeutic agent to provide superior renal protection, due to its strong antioxidant activity and ability to protect damaged the renal epithelial cell based on the mitochondrial pathway.

## References

[1] Hasui M., Matsuda M., Okutani K., Shigeta S. (1995). *In vitro* antiviral activities of sulfated polysaccharides from a marine microalga (*Cochlodinium polykrikoides*) against human immunodeficiency virus and other enveloped viruses. Int. J. Biol. Macromol..

[2] Karnjanapratum S., You S. (2010). Molecular characteristics of sulfated polysaccharides from *Monostroma nitidum* and their *in vitro* anticancer and immunomodulatory activities. Int. J. Biol. Macromol..

[3] Rajeswari A., Varalakshmi P. (2006). Low molecular weight heparin protection against oxalate-induced oxidative renal insult. Clin. Chim. Acta.

[4] Wijesekara I., Pangestuti R., Kim S.K. (2011). Biological activities and potential health benefits of sulfated polysaccharides derived from marine algae. Carbohydr. Polym..

[5] Zhang Z., Zhang Q., Wang J., Zhang H., Niu X., Li P. (2009). Preparation of the different derivatives of the low-molecular-weight porphyran from *Porphyra haitanensis* and their antioxidant activities *in vitro*. Int. J. Biol. Macromol..

[6] Kardošová A., Machová E. (2006). Antioxidant activity of medicinal plant polysaccharides. Fitoterapia.

[7] Borghi L., Meschi T., Guerra A., Bergamaschi E., Mutti A., Novarini A. (1995). Effects of urinary macromolecules on the nucleation of calcium oxalate in idiopathic stone formers and healthy controls. Clin. Chim. Acta.

[8] Edyvane K.A., Hibberd C.M., Harnett R.M., Marshall V.R., Ryall R.L. (1987). Macromolecules inhibit calcium oxalate crystal growth and aggregation in whole human urine. Clin. Chim. Acta.

[9] Liu J., Wang T., Chen J., Wang S., Ye Z. (2006). Decreased inhibitory activity of prothrombin to calcium oxalate crystallization by specific chemical modification of its gamma-carboxyglutamic acid residues. Urology.

[10] Hackett R.L., Shevock P.N., Khan S.R. (1994). Madin-Darby canine kidney cells are injured by exposure to oxalate and to calcium oxalate crystals. Urol. Res..

[11] Koul H., Kenington L., Honeyman T., Jonassen J., Menon M., Scheid C.R. (1996). Activation of the c-myc gene mediates the mitogenic effects of Ox in LLC-PK1 cells, a line of renal epithelial cells. Kidney Int..

[12] Bashir S., Gilani A.H. (2009). Antiurolithic effect of *Bergenia ligulata* rhizome: An explanation of the underlying mechanisms. J. Ethnopharmacol..

[13] Niimi K., Yasui T., Hirose M., Hamamoto S., Itoh Y., Okada A., Kubota Y., Kojima Y., Tozawa K., Sasaki S. (2012). Mitochondrial permeability transition pore opening induces the initial process of renal calcium crystallization. Free Radic. Biol. Med..

[14] Cao L.C., Honeyman T.W., Cooney R., Kennington L., Scheid C.R., Jonassen J.A. (2004). Mitochondrial dysfunction is a primary event in renal cell oxalate toxicity. Kidney Int..

[15] Zoratti M., Szabò I. (1995). The mitochondrial permeability transition. Biochim. Biophys. Acta Rev. Biomembr..

[16] Veena C.K., Josephine A., Preetha S.P., Rajesh N.G., Varalakshmi P. (2008). Mitochondrial dysfunction in an animal model of hyperoxaluria: A prophylactic approach with fucoidan. Eur. J. Pharmacol..

[17] Wang J., Zhang Q.B., Zhang Z.S., Zhang H., Niu X.Z. (2010). Structural studies on a novel fucogalactan sulfate extracted from the brown seaweed *Laminaria japonica*. Int. J. Biol. Macromol..

[18] Samee H., Li Z.X., Lin H., Khalid J., Wang B.P. (2009). *In vivo* study of antiallergenicity of ethanol extracts from *Sargassum tenerrimum*, *Sargassum cervicorne* and *Sargassum graminifolium turn*. Eur. Food Res. Technol..

[19] Zhang C.-Y., Wu W.-H., Lan M.-B. (2012). Antioxidant properties of polysaccharide from the brown Seaweed *Sargassum graminifolium (Turn.)*, and its effects on calcium oxalate crystallization. Mar. Drugs.

[20] Bubber P., Ke Z.-J., Gibson G.E. (2004). Tricarboxylic acid cycle enzymes following thiamine deficiency. Neurochem. Int..

[21] Küçükkurt I., Ince S., Keles H., Akkol E.K., Avci G., Yesilada E., Bacak E. (2010). Beneficial effects of *Aesculus hippocastanum* L. seed extract on the body’s own antioxidant defense system on subacute administration. J. Ethnopharmacol..

[22] Srinivasan S., Pragasam V., Jenita X., Kalaiselvi P., Muthu V., Varalakshmi P. (2004). Oxidative stress in urogenital tuberculosis patients: A predisposing factor for renal stone formation amelioration by vitamin E supplementation. Clin. Chim. Acta.

[23] Muthukumar A., Selvam R. (1998). Role of glutathione on renal mitochondrial status in Hyperoxaluria. Mol. Cell. Biochem..

[24] Chaiyarit S., Thongboonkerd V. (2012). Changes in mitochondrial proteome of renal tubular cells induced by calcium oxalate monohydrate crystal adhesion and internalization are related to mitochondrial dysfunction. J. Proteome Res..

[25] Veena C.K., Josephine A., Preetha S.P., Varalakshmi P. (2007). Beneficial role of sulfated polysaccharides from edible seaweed *Fucus vesiculosus* in experimental hyperoxaluria. Food Chem..

[26] Bayir Y., Halici Z., Keles M.S., Colak S., Cakir A., Kaya Y., Akçay F. (2011). *Helichrysum plicatum* DC. subsp. *plicatum* extract as a preventive agent in experimentally induced urolithiasis model. J. Ethnopharmacol..

